# Induction of Tolerance to Therapeutic Proteins With Antigen-Processing Independent T Cell Epitopes: Controlling Immune Responses to Biologics

**DOI:** 10.3389/fimmu.2021.742695

**Published:** 2021-09-09

**Authors:** Evelien Schurgers, David C. Wraith

**Affiliations:** ^1^Apitope International NV, Diepenbeek, Belgium; ^2^Institute of Immunology and Immunotherapy, University of Birmingham, Birmingham, United Kingdom

**Keywords:** Immunological tolerance, haemophilia A, Tr1 cell, Treg cell, immunotherapy, hypersensitivity, synthetic peptide, T cell epitope

## Abstract

The immune response to exogenous proteins can overcome the therapeutic benefits of immunotherapies and hamper the treatment of protein replacement therapies. One clear example of this is haemophilia A resulting from deleterious mutations in the FVIII gene. Replacement with serum derived or recombinant FVIII protein can cause anti-drug antibodies in 20-50% of individuals treated. The resulting inhibitor antibodies override the benefit of treatment and, at best, make life unpredictable for those treated. The only way to overcome the inhibitor issue is to reinstate immunological tolerance to the administered protein. Here we compare the various approaches that have been tested and focus on the use of antigen-processing independent T cell epitopes (apitopes) for tolerance induction. Apitopes are readily designed from any protein whether this is derived from a clotting factor, enzyme replacement therapy, gene therapy or therapeutic antibody.

## Introduction

The last 30 years has seen an upsurge in the use of drugs produced using biological systems rather than chemical synthesis (biologics) for treatment of cancer and inflammatory diseases. This is such that in 2019, biologics, largely monoclonal antibodies and fusion proteins, constituted 73% of major drug sales. This reflects the high specificity of such agents for their disease targets. The disadvantage of large exogenous proteins as therapeutics, however, is their immunogenicity. While the incidence of immunogenicity is reduced by ‘humanisation’ of monoclonal antibodies, anti-drug antibodies frequently arise following repeated administration. For example, the ABIRISK consortium found antibodies to the most popular anti-TNF agent (Humira) increased in frequency with time reaching ~50% at 18 months (1). A similar issue affects people with haemophilia A or B, conditions caused by deficiency in or production of defective clotting factors VIII or IX respectively (2). A recent study showed that recombinant FVIII injections, designed to provide effective levels of clotting factor, induce anti-FVIII antibodies (‘inhibitors’) at a higher frequency than factor purified from plasma (3). The cumulative incidence of inhibitors was 26.8% with plasma derived FVIII and 44.5% with the more frequently used recombinant FVIII.

FVIII inhibitors arise in people with altered FVIII genes who fail to produce a fully functional protein. In unaffected individuals, immunological tolerance to FVIII is generated by both central and peripheral tolerance mechanisms. The lack of complete protein in haemophiliacs results in ineffective tolerance and leads to susceptibility to inhibitor formation. FVIII-specific immune activation occurs through antigen-presenting cells (APC) that internalize the FVIII protein, process and present antigenic peptides (epitopes) on major histocompatibility complex class II (MHCII) molecules to CD4^+^ T-cells in the presence of co-stimulatory signals (4). Activation of naïve B and CD4^+^ T helper cells, including follicular helper T cells, is subsequently amplified by B-T collaboration resulting in affinity maturation and switching from IgM to IgG isotypes. FVIII inhibitors are CD4^+^ T-cell-dependent in both murine haemophilia models (5–7) and HA patients (8) and are of high affinity i.e. B cells producing them have undergone affinity maturation. Recent studies have shown that FVIII-specific antibodies in patients with inhibitors have up to a 100-fold higher apparent affinity compared to antibodies in patients without inhibitors or healthy individuals (9). The inference from these observations is that it should be possible to control affinity maturation and inhibitor formation by induction of tolerance in FVIII-specific CD4^+^ T cells.

## Inducing Tolerance to FVIII

Approaches to induce tolerance to FVIII were reviewed recently by Lacroix-Demazes and colleagues (10). Repeated high doses of FVIII through immune tolerance induction (ITI) dampens the immune response in many but not all recipients; however, it is burdensome on patients and so expensive (11) that it is unavailable in many countries. There is a critical need for more effective and less expensive approaches to tolerance induction.

Various broad acting immunosuppressive approaches have been tested. These include co-administration of FVIII with rapamycin (12), co-stimulatory pathway modulators (13) and agents to selectively deplete B cell subsets (14). More directly antigen-specific approaches have been tested to improve the efficacy of ITI. For example, coupling FVIII to the Fc portion of immunoglobulin targets the protein to B cells *via* inhibitory Fc receptors. Both pre-clinical and early clinical studies show that this reduces inhibitor formation (15). A promising approach is to modify Foxp3^+^ regulatory T (Treg) cells with T cell receptors specific for FVIII epitopes or a single chain Fv specific for FVIII protein (16, 17). Foxp3^+^ Treg cells were shown to mediate linked suppression whereby Treg cells specific for a single epitope would suppress the immune response to other epitopes within the protein. This approach would involve complex modification of the patient’s own Treg cells; furthermore, it is not clear how frequently Treg infusions would be required and, therefore, how practical this would be in the clinic.

An alternative approach is to administer antigens in a form that selectively induces Treg cells *in vivo*. For example, it has long been known that mucosal delivery of antigen can induce suppression through various mechanisms of tolerance including apoptosis, anergy and Treg induction (18). Herzog and colleagues have shown that FVIII fused to the B-subunit of cholera toxin B and encapsulated in plant cells can control inhibitor formation when given repeatedly by oral administration to mice (19). Oral tolerance correlated with an increase of immunoregulatory cytokines, IL-10 and TGF-β, with upregulation of the Foxp3 gene in antigen-specific cells.

Various nanoparticle-based approaches for suppression of inhibitors have been tested. Encapsulation of FVIII in phosphatidyl serine enriched liposomes targets the protein to tolerogenic APC capable of inducing Treg cells. Treatment of mice led to suppression of inhibitor antibody formation associated with an increase in Foxp3^+^ Treg cells (20). As reviewed elsewhere (21), both macrophage and dendritic cell populations can have tolerogenic properties *in vivo*. Consequently, antigen-specific tolerance can be induced by administration of antigens linked to antibodies against receptors on steady state dendritic cells thus proving their tolerogenic potential (22). Similarly, nanoparticles containing immunosuppressive drugs such as rapamycin can be used to suppress the immune response to co-administered FVIII (23). The rapamycin study is especially interesting because the treatment was successful when rapamycin nanoparticles were injected alongside treatment with FVIII, the drug and antigen did not need to be in the same particle. This implies that the nanoparticles can promote systemic immune modulation resulting in the maintenance of tolerance to FVIII.

## Immunotherapy in the Face of a Pandemic

The world is currently facing the challenge of COVID-19. Not since the 1918 HINI Influenza pandemic has the human race faced such a threat from an infectious agent. This has brought immunotherapy and the use of non-specific immune modulating drugs into sharp focus. Many patients with cancer or chronic inflammatory conditions are experiencing long periods of isolation as they wait for the rest of the population to benefit from vaccination and the pandemic to recede. Clearly, we must find more selective ways to treat such conditions so that the immune system remains intact both to combat such infections and respond effectively to vaccination. Many of the approaches mentioned above fall short of an effective immunotherapy. Any approach depleting B or T cells or non-specifically modulating the function of APC will leave the individual immune compromised. We must strive for antigen-specific approaches that induce tolerance to therapeutic proteins but do not compromise the rest of the immune system. Here we will review our experience with apitopes. Apitopes are antigen processing independent T cell epitopes that selectively induce tolerance among CD4^+^ T cells.

## Mechanism of Action of Apitopes

Apitopes are CD4 T-cell epitopes designed to bind directly to their MHC II restriction element in the correct conformation for recognition by their cognate T cell (24). These peptides are identical to the native protein but may be modified to improve solubility by addition of hydrophilic amino acids at N- and C-termini. As reviewed elsewhere (21), apitopes differ from other forms of peptide therapy since they do not depend on combination with cells or nanoparticles or chemical modification for their function. Our path towards developing apitopes for treatment of autoimmune and allergic conditions began with an investigation of mucosal tolerance (25). This showed that while oral administration of protein was unreliable as a means of inducing tolerance, high doses of peptide could be effective. However, it became clear that the oral route was relatively ineffective when compared with the intranasal route for peptide antigens as a result of the degradative nature of the gut (26). Later work revealed that subcutaneous delivery of soluble peptides was far more effective than either of the two mucosal routes (27). Initial studies showed that some known T cell epitopes were tolerogenic while others were not (28). Our work subsequently revealed that tolerogenic T cell epitopes bind directly to MHC II in a conformation that mimics the naturally processed epitope whereas non-tolerogenic epitopes either bind to MHC molecules in a cryptic conformation (29) or were insufficiently soluble to induce tolerance (30). Tolerogenic CD4 T cell epitopes are, therefore, designed as apitopes that bind directly to MHC class II molecules in the correct conformation and are sufficiently soluble to induce tolerance. We recently revealed that soluble apitopes selectively bind to steady state dendritic cells and not B cells or monocytes in lymphoid organs (24). Steady state dendritic cells are tolerogenic because they express low levels of costimulatory molecules and, importantly, selectively bind apitopes because they have peptide receptive MHC II at the cell surface (31).

**Figure 1:**
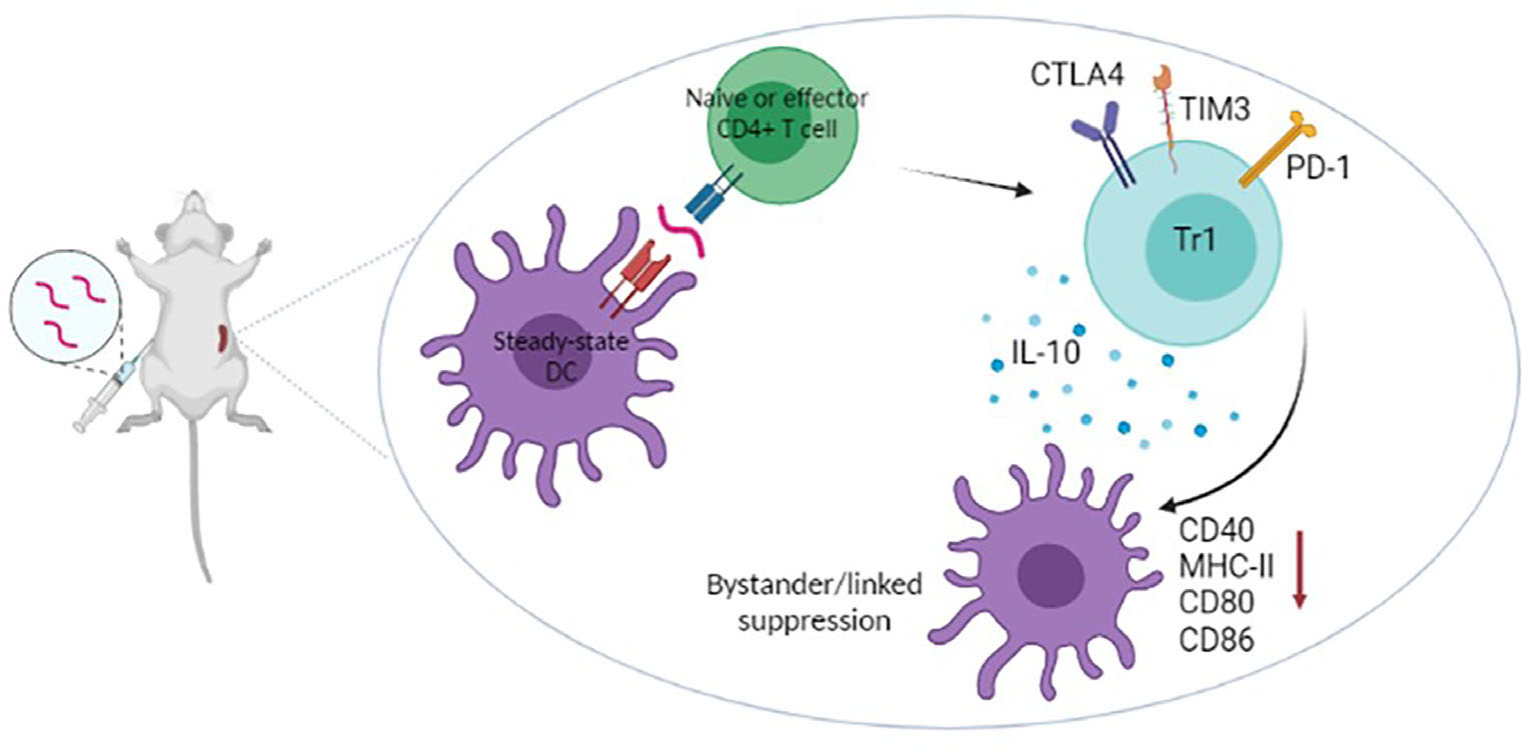
Recent work has shown that apitopes injected subcutaneously migrate rapidly to (<5mins) and bind MHC II on steady-state DC in lymphoid organs (24). Naïve cells undergo abortive activation then, on repeated dosing, develop anergy and upregulate genes characteristic of Tr1 cells (IL-10 and inhibitory receptors) (27, 32). Tr1 cells specific for epitope A within an antigen cause downregulation of the antigen presenting machinery of antigen presenting cells (33) hence blocking their ability to present epitopes B,C,D etc from the same or associated antigens. Apitope induced Tr1 cells mediate both linked and bystander suppression.

Presentation of peptide antigens on steady state dendritic cells (**Figure 1**) increases the proportion of Foxp3^+^ T cells *in vivo* but the major impact of treatment with apitopes is to convert potentially pathogenic T cells into regulatory Tr1 cells (27, 34, 35). These Tr1 cells are anergic, IL-10 producing cells that are Foxp3^-^ but express a similar tolerance associated gene signature to the IL-10 producing cells that control immune pathology in chronic infection (36). In particular, they express the IL-10 promoting transcription factors c-Maf and NFIL3 and upregulate inhibitory receptors CTLA-4, LAG-3, TIM-3 and TIGIT (27). Recently, through analysis of T-cell receptor signalling, epigenetic modification and gene expression, we have shown that the induction of Tr1 cells involves suppression of both signalling to and chromatin priming of immune response genes. At the same time, chromatin priming of those genes associated with the Tr1 cell tolerance signature was promoted making these genes sensitive to levels of signalling below the threshold needed to activate immune response genes (32). This study explains how repeated encounter of antigen, in the form of apitopes presented by steady state dendritic cells, prevents the differentiation of pathogenic/effector cells but leads to dominant tolerance through generation of a regulatory Tr1 population.

## Development of Apitopes for Treatment of Autoimmune Diseases

Apitopes can be used to suppress immune pathology in response to biologics, allergies and autoimmune diseases. The most advanced clinical programmes are for autoimmune diseases including multiple sclerosis (MS) and Graves’ disease (GD). Our experience with these programmes informs the use of apitopes for aberrant responses to biologics.

Graves’ disease is caused by the generation of autoantibodies specific for the thyroid stimulating hormone receptor (TSHR). These antibodies cause chronic activation of the receptor leading to excessive secretion of thyroid hormones and hyperthyroid disease. Apitope designed 2 peptides (5DK and 9B) from TSHR that were pan-DR binding, highly soluble and induced tolerance to the TSHR in a relevant HLA-DR3 transgenic mouse model (37). Apitope designs pan-DR epitopes primarily because Graves’ is associated with different MHC haplotypes; therefore, while most Caucasian Graves’ patients are HLA-DR3^+^, the link to a specific HLA-DR is less clear in Asian populations. It is important to design peptides with the capacity to bind to and be recognised by people with a broad range of HLA-DR types. We have shown, for example, that peptide 5DK will suppress the response to TSHR in both HLA-DR3 and HLA-DR4 transgenic mice.

Based on our analysis of the mechanism of action of model apitope peptides, we have designed an ‘in patient’ dose escalation protocol that optimises the generation of Treg cells (27, 32). Patients are given either a subcutaneous or intradermal dose of peptide increasing from 25 to 800 µg of the peptide cocktail every 2 weeks. In the Graves’ phase 1 study this resulted in a reduction in anti-TSHR antibody levels that correlated with a return of thyroid hormone levels to the normal range in 7 of 10 patients treated (38).

It is believed that MS is driven by the immune response to a range of different myelin antigens including myelin basic protein (MBP), proteolipid protein and myelin oligodendrocyte protein. We identified 4 dominant epitopes from MBP that could be designed as apitopes. The cocktail of 4 MBP apitopes (ATX-MS-1467) induced tolerance and promoted IL-10 secreting T cells in HLA-DR2 transgenic mice (39, 40). Two phase 1 trials showed that intradermal ATX-MS-1467 was safe with evidence of efficacy in patients with secondary progressive (39) and relapsing MS (41). A phase 2 study in relapsing MS revealed that ATX-MS-1467 not only suppressed CNS inflammation but significantly improved cognition in treated patients (41). The implication from these early trials in Graves’ disease and MS is that selected T cell epitopes from within one autoantigen can control both the response to the whole autoantigen (linked suppression) and the response to other antigens within the same tissue (bystander suppression). Linked and bystander suppression mediated by antigen-specific immunotherapy with peptide epitopes has been described previously in experimental animal models (28, 35, 42).

## Design of Apitopes From FVIII

The nature of the immune response to FVIII was reviewed recently by Varthaman and Lacroix-Desmazes (43). They emphasise the key role of CD4^+^ T cells in supporting generation of inhibitor antibodies and summarise previous work on defining the immunodominant T cell epitopes driving the response. Although some previous studies have indicated a link with the HLA-DR type of people developing inhibitors (44) this has not been substantiated. For this reason, our approach has been to identify pan-DR binding epitopes expected to function in a wide range of the population. The combined use of various MHC-binding algorithms identified a set of 12 peptides that were then used to screen responses among human peripheral blood mononuclear cells and T cells from FVIII-immune HLA-DR transgenic mice (45). Two dominant T cell epitopes were identified spanning amino acids 545-559 and 1788-1802. Importantly, an independent analysis by Steinitz and co-workers, using a screen of overlapping peptides from the whole FVIII protein, identified the same peptides among 3 immunodominant peptides arising in HLA-DR transgenic mice immunised with Baxter’s recombinant FVIII (46). In a separate study, van Haren undertook an elegant experiment whereby they allowed dendritic cells from a range of donors to process FVIII protein (47). They then eluted peptides from HLA-DR and identified the peptides by mass spectrometry. The dominant epitope identified by this alternative approach contained the FRNQASRPY sequence found within our 1788-1802 epitope. Importantly, this peptide was found in dendritic cells from a heterogeneous group of donors with a broad range of HLA-DR types hence confirming its pan-DR binding characteristics.

Peptides 545-559 and 1788-1802 were optimised for solubility by addition of lysine residues at the N- and C-termini to create apitopes P1 and P17 respectively. A combination of P1 and 17 (ATX-F8-117) was injected subcutaneously in saline in HLA-DR transgenic mice. This resulted in suppression of the immune response to FVIII as evidenced by inhibition of the T cell proliferative response to FVIII *in vitro* with cells from mice immunised with FVIII in Freund’s adjuvant. Furthermore, mice treated with either peptide alone showed a suppressed immune response to FVIII. HLA-DR transgenic mice injected with 1 µg FVIII in saline solution weekly develop anti-FVIII antibodies after the 4^th^ subcutaneous injection (46). HLA-DR transgenic mice were treated with ATX-F8-117 using a dose escalation protocol prior to repeated challenge with FVIII in saline. This significantly inhibited the generation of both total anti-FVIII and inhibitor antibodies (45). Furthermore, we have shown that treatment with ATX-F8-117 suppresses new inhibitor formation in mice previously immunised with FVIII. Importantly, FVIII inhibitor levels fell away from the time at which the apitope treatment was started. This promising result shows that dominant T cell epitopes from FVIII can be used to both prevent and reverse inhibitor formation in HLA-transgenic mice. ATX-F8-117 warrants investigation in phase 1 trials of immunotherapy in people with inhibitors.

## Discussion

The central role of CD4 T cells in orchestrating the immune response in allergy, autoimmune disease and the antigen-drug antibody response to biologics is clear. For example, haemophiliacs with a history of inhibitors who became HIV positive failed to make an anamnestic response to subsequent FVIII infusions (48). Furthermore, inhibition of CD4 T cell priming by blockade of costimulatory pathways prevented the generation of inhibitors in a mouse model of haemophilia A (49). Reinstating immunological tolerance to specific antigens through antigen-specific immunotherapy is designed to suppress the immune pathology associated with hypersensitivity conditions while leaving the immune system of an individual capable of protecting against infectious diseases, of responding to vaccination or immune surveillance of cancers. As discussed above, there is an urgent need to move away from the use of immune debilitating drugs for hypersensitivity conditions especially when faced with the threat of emerging infections and global pandemics.

Various approaches to antigen-specific immunotherapy are under development. These include the use of nanoparticles designed to target antigens to the liver or to tolerogenic APC in lymphoid organs (21). A recent advance described the use of an RNA vaccine to deliver a peptide in a tolerogenic form to treat a mouse model of MS (50). This approach would have clear advantages if it were possible to deliver an intact protein in a tolerogenic fashion.

As it stands, the most straightforward approach to antigen-specific immunotherapy is the design of tolerogenic peptide epitopes. Our work on this topic was first described in 1989 when we realised that a high affinity analogue of a T cell epitope could be used to switch off the immune response *in vivo* (51). Subsequent work showed that high affinity analogues in adjuvant caused activation induced cell death among responsive cells (52) and led to investigation of peptide delivery without adjuvants. Over the past two decades we have defined the rules governing successful tolerance induction with T cell epitopes (**Table 1**). Key features are that the peptides need to be designed as antigen processing independent epitopes (apitopes) (29), need to be highly soluble so as to reach steady state/tolerogenic dendritic cells in lymphoid organs (24), can be made more potent by optimising affinity for MHC (54) and are capable of both linked and bystander suppression. The ability of a single T cell epitope from a large, complex protein such as FVIII to suppress the response to the whole protein has been demonstrated in our recent study (45). This important observation emphasises the impact of linked suppression for both prevention of and suppression of ongoing immune responses.

**Table 1:** Properties of antigen processing independent T cell epitopes (apitopes) designed for antigen-specific immunotherapy.

Apitope Property	Comment	References
**Preferred route of administration**	Intradermal/subcutaneous > intranasal >> oral	(25–27)
**MHC II binding**	Apitopes must bind MHC II in a conformation that mimics the naturally processed antigenic epitope	(29)
***In vivo* interaction with antigen presenting cells**	Apitopes bind selectively to steady-state dendritic cells rather than B cells or monocytes	(24)
**Solubility**	Apitopes must be soluble such that they rapidly migrate in the fluid phase to steady-state dendritic cells in lymphoid organs following subcutaneous administration	(24)
**Induction of regulatory T cells in mice**	Repeated administration of the model apitope Ac1-9[4Y] induces both Foxp3^+^ Treg and Foxp3^-^ Tr1 cells (Tr1 >> Foxp3^+^ Treg)	(27, 33–35, 53)
**Clinical development (Graves’ disease)**	Phase 1 trial of two peptides from the thyroid stimulating hormone receptor in patients with mild to moderate hyperthyroid disease showed a treatment related response in 7 of 10 individuals	(37, 38)
**Clinical development (Multiple Sclerosis)**	Phase 1 trials in secondary progressive and relapsing MS (RMS) using a cocktail of four peptides from myelin basic protein revealed no unexpected safety signals and evidence of efficacy (significant reduction of enhancing lesions) in RMS patients. Phase 2 demonstrated a similar reduction in lesion volume with improvement in cognition in RMS patients	(39, 41)
**Clinical development (Inhibitor formation in haemophilia A)**	Two pan-DR binding peptides from factor VIII were optimised for solubility and shown to either prevent the generation of or suppress levels of inhibitors in HLA-DR transgenic mice, previously immunised with FVIII, when the apitopes were injected subcutaneously	(45)

The fact that simple apitopes designed from antigens, including self-antigens such as TSHR (37, 38) and exogenous antigens such as FVIII (45), can control pathogenic antibody responses to these proteins provides evidence that they can be readily designed for any antibody-mediated complication where the antigen is known. This includes other autoimmune conditions such as myasthenia gravis, allergic conditions and the aberrant immune response to biologics or enzyme replacement therapies. Our work has shown that it is now possible to control hypersensitivity conditions through antigen-specific immunotherapy thereby avoiding the use of non-specific immunosuppressive drugs.

## Author Contributions

All authors listed have made a substantial, direct, and intellectual contribution to the work and approved it for publication.

## Conflict of Interest

DW is CSO and Founder of Apitope International NV. ES is an employee of Apitope International NV.

## Publisher’s Note

All claims expressed in this article are solely those of the authors and do not necessarily represent those of their affiliated organizations, or those of the publisher, the editors and the reviewers. Any product that may be evaluated in this article, or claim that may be made by its manufacturer, is not guaranteed or endorsed by the publisher.
